# Effectiveness of take ACTION online naloxone training for law enforcement officers

**DOI:** 10.1186/s40352-023-00250-9

**Published:** 2023-11-18

**Authors:** Chin Hwa Dahlem, Rohan Patil, Lara Khadr, Robert J. Ploutz-Snyder, Carol J. Boyd, Clayton J. Shuman

**Affiliations:** 1 Center for the Study of Drugs, Alcohol, Smoking and Health, Department of Health Behavior and Biological Sciences, School of Nursing, University of Michigan, 400 N. Ingalls Rd Rm 3174, Ann Arbor, MI 48109 USA; 2 Applied Biostatistics Laboratory, Ann Arbor, USA; 3 Department of System Populations and Leadership, Ann Arbor, USA; 4 Institute for Healthcare Policy and Innovation, University of Michigan, Ann Arbor, USA

**Keywords:** Law enforcement, Naloxone, Overdose, Police, Online, Attitudes, Knowledge

## Abstract

**Background:**

Training law enforcement officers (LEOs) to administer naloxone is a recommended strategy to reduce overdose deaths in the United States. To achieve this, an evidence-based and scalable naloxone training curriculum that is easy to use and readily scalable is needed. Convenient web-based training is a flexible method for delivering educational interventions particularly for LEOs who have irregular or shifting schedules. This study examined the effectiveness of a comprehensive web-based naloxone training that was created in partnership with LEOs on their knowledge, confidence, and attitudes regarding naloxone.

**Methods:**

From May 2019 to September 2020, five law enforcement departments from Michigan participated in web-based naloxone training. A total of 182 LEOs (77% male) were in the final sample based on matching pre-and post-test surveys. LEOs were assessed on knowledge, confidence, and attitudes towards naloxone. Negative binomial and Poisson regression was conducted to assess associations between knowledge, confidence, and attitudes towards naloxone before and after training.

**Results:**

Significant improvements in overdose knowledge and confidence were revealed across all departments with median (IQR) total composite scores for knowledge increasing from 35 (32, 37) to 40 (39, 42) (p < 0.01) and confidence increasing from 18.5 (15, 20) to 20 (20, 25) (p < 0.01). Median (IQR) attitude scores did not change.

**Conclusion:**

Our web-based naloxone training was effective in improving knowledge and confidence for LEOs but did not significantly improve LEOs attitudes towards naloxone across most departments. The web-based format is readily scalable and quickly disseminated and meets the immediate need for LEO overdose training. Additional intervention is needed to address the negative attitudes of LEOs regarding naloxone.

**Supplementary Information:**

The online version contains supplementary material available at 10.1186/s40352-023-00250-9.

## Background

Drug overdose deaths continue to be a significant cause of mortality and have increased for nearly two decades in United States (Hedegaard et al., 2021; Ahmad et al., 2023). In 2021 alone, over 107,000 drug overdose deaths occurred, with 78% of these deaths attributed to opioids, predominantly synthetic (fentanyl) opioids (Spencer et al., 2022). Moreover, deaths involving stimulants, such as cocaine and methamphetamine, have risen in part due to co-involvement with opioids (Kariisa et al., 2021).

In response to the rising number of overdose deaths, several strategies to prevent opioid overdoses have been recommended and implemented in the United States (Davis et al., 2014). As of March 2014, federal organizations, including the Department of Justice (*Attorney General Holder Announces Plans for Federal Law Enforcement Personnel to Begin Carrying Naloxone*, 2014), recommend that law enforcement officers (LEOs) be trained and equipped to respond to overdoses with naloxone, an opioid agonist that can be safely administered by LEOs (Dahlem et al., 2017; Davis et al., 2015; Fisher et al., 2016; McCarthy et al., 2019; Wagner et al., 2016). With the passing of legislation requiring LEOs to be trained and equipped to administer naloxone throughout many states (Davis & Carr, 2015), 2,340 law enforcement agencies in 42 states have been prepared to respond to overdoses as of March 2018 (Lurigio et al., 2018). Despite this, in a recent survey of 2,829 LEOs working across 20 states, 15% of the LEOs reported their departments do not regularly carry naloxone while on patrol (Carroll et al., 2020). Furthermore, the LEOs who previously responded to overdoses identified the need for additional training regarding safe handling of opioids, states’ 911 Good Samaritan Law, and identifying overdose symptoms (Carroll et al., 2020).

Studies have shown that LEOs overdose knowledge (Nath et al., 2020; Saucier et al., 2016; Wagner et al., 2016) and attitudes (Purviance et al., 2017; Ray et al., 2015) improve after receiving naloxone training; moreover, LEOs can safely administer naloxone (Dahlem et al., 2017; Fisher et al., 2016; Jacoby et al., 2020) and equipping first responders with naloxone is associated with decreased overdose deaths (Rando et al., 2015). However, there are studies showing LEOs attitudes towards overdose survivors are worse (Winograd et al., 2020a) or attitudes about overdose survivors do not change after the naloxone training (Wagner et al., 2016). In addition, with concerns about naloxone access leading to increased drug use (Green et al., 2013; Burris et al., 2009) or riskier drug use (Reichert et al., 2019) despite empirical evidence, these concerns exist and could be addressed in overdose prevention curriculum (Winograd et al., 2020b). In fact, in a scoping review of law enforcement naloxone training, most programs covered elements of harm reduction goals, overdose recognition and response, occupational safety, and policing practices, but gaps remain with regard to training development, design, evaluation, and content related to facilitating interactions with people who use drugs (PWUD) and stigma reduction (Khorasheh et al., 2019).

As law enforcement are often first to arrive at the scene of an overdose (Davis et al., 2014; White et al., 2022; Pourtaher et al., 2022) and with 2,300 law enforcement agencies (Lurigio et al., 2018) throughout United States, it is imperative that naloxone trainings for LEOs be readily available, broadly disseminated, and easily implemented by law enforcement agencies. Prior training efforts using in-person trainers have been generally one hour long, power point presentations that were incorporated with in service training (Khorasheh et al., 2019). Challenges to implementation for in person trainings include costs associated with in-person training (Dahlem et al., 2022), availability of trainers across different shifts (e.g., day shift, night shift), staffing, legal issues, organizing an entire department to receive training with limited time availability (Silverman et al., 2012), and regulatory concerns (Salvador et al., 2020; Winstanley et al., 2016). Thus, one strategy to address several of these barriers is to use a web-based format for training. Web-based learning provides a more conducive, accessible learning environment while maintaining the quality and effectiveness of the training (Hill et al., 2021). Online training is readily scalable, having the advantage of reaching rural areas and states experiencing high opioid related mortality (Simmons et al., 2022).

Although a plethora of naloxone training exists for first responders and other community laypeople, few have examined the direct effects of LEO training on overdose knowledge, confidence, and attitudes about naloxone via web-based training format. Simmons et al. (2016) examined the feasibility and acceptability of an online training for first responders and found that first responders were highly satisfied with the content, format, and mode of delivery, and were confident and prepared to intervene in overdose emergencies (Simmons et al., 2016). However, the authors were not able to adequately assess knowledge gained nor attitudinal shifts due to the training. Dunn et al. (2017), with a sample of substance users, demonstrated that participants who were undergoing detoxification had significant and sustained increases in overdose knowledge and reductions in high-risk behaviors after receiving a computerized overdose educational intervention versus education delivered via written pamphlet (Dunn et al., 2017).

Our strategy delivers a web-based naloxone training program for LEOs to efficiently train them to respond to opioid overdoses. Our Take ACTION program was developed and validated in partnership with our community partners (e.g., local Sheriff’s Office and Substance Use Disorder treatment provider). To our knowledge, no study has evaluated the direct effects of a web-based naloxone training for LEOs on knowledge, confidence, and attitudes about naloxone and PWUD. Therefore, the purpose of this study was to determine the effectiveness of a web-based naloxone training on opioid overdose knowledge, confidence, and attitudes on a larger sample of LEOs.

## Methods

### Design

This study was a nonrandomized pre-and post-test survey evaluation of LEOs who participated in a web-based naloxone training as part of their mandatory education requirement to be equipped with naloxone when out in the field.

### Sample and setting

Five law enforcement agencies from Michigan were recruited to use the web-based training for their LEOs from May 2019 to September 2020 based on first author’s prior relationships with the agencies. Two of the law enforcement agencies were city police departments and three were county police agencies. All five law enforcement agencies were in urban counties in [Midwest State] in which opioid overdose death rates per 100,000 residents ranged from 21.5 to 37 (Michigan Overdose Data, 2022). The web-based naloxone training was offered for each of the agencies to train their new and current LEOs.

### Intervention

The detailed development of the web-based naloxone training using community engaged research methods with the Sheriff’s Office and a local substance use treatment center and its feasibility and acceptability assessment is published elsewhere (Dahlem et al., 2023). Initial content development for the web-based curriculum was based on our initial in person PowerPoint assisted naloxone training that included the essential elements of overdose response and recognition, addressing myths and facts about naloxone and overdoses, protocols for post-administration referral processes, and the testimony of person in recovery (Dahlem et al., 2017). Through qualitative interviews of novice and experienced LEOs and meetings with our community advisory team, modifications of the web-based content occurred (Dahlem et al., 2022, 2023).

The web-based course consisted of short self-paced modules that incorporated multimedia videos and animation along with a pre-and post-test evaluation. The module topics included: (1) epidemiology of the opioid epidemic, (2) opioid legislation, (3) opioid pharmacology including fentanyl and fears associated with incidental contact and overdoses, (4) risk factors, (5) overdose response, (6) post-overdose care, and (7) stigma reduction through discussion of myths and facts surrounding naloxone, addiction, and overdoses by real LEOs and people in long-term recovery. The additional feature of certificate of completion was added so that LEOs who completed the training would be able to submit to their agencies that the training was completed.

Three short videos, less than 4 min each, were created to show how to respond to an opioid overdose and administer naloxone, a roundtable of active LEOs discussing their experiences with using naloxone and addressing fears LEOs may have with naloxone and overdoses, and a roundtable of people in long-term recovery addressing misconceptions about naloxone and drug use. The misconceptions discussed by LEOs included: (1) We will arrest the overdose victim or the person who called for help, because they were using drugs, (2) the person who receives naloxone will react violently when the medication is administered and his/her opioid overdose is reversed, (3) There is very little you can do when a person is having an opioid overdose since he/she can die instantaneously, (4) Since an overdose is a medical issue, EMS should respond rather than law enforcement. For people in long-term recovery, misconceptions addressed were: (1) It is really hard to prevent a person dying from a drug overdose since people usually use drugs in private, (2) Using naloxone will delay a person’s entry into drug treatment and encourage even more risky drug use, (3) It is a waste of resources to save a person with naloxone since he or she is likely to overdose again and again. All content and script for the videos were reviewed by the PI, LEOs, people in recovery, and experts in overdose education (Dahlem et al., 2023). The videos can be accessed through the online training or separately viewed on www.overdoseaction.org.

The median time to complete the web-based naloxone training including the pre-and post-tests was 45 min (range of 37–80 min). For the LEOs in this study, the web-based naloxone training was mandatory.

### Data collection procedures

When registering for the web-based naloxone training, each LEO was provided introductory information on what to expect for the training and their voluntary consent to use the survey data collected to facilitate our understanding of effectiveness of the web-based training. All pre- and post-test surveys were embedded into the naloxone training. This study was considered exempt by a University of Michigan Institutional Review Board and considered no more than minimal risk.

### Measures

We collected LEO demographics including self-reported age, gender, race/ethnicity, and organization. We also asked whether they received naloxone training before (yes/no), if they have used naloxone previously (yes/no), the number of times naloxone was used if applicable, and multiple-choice option of why they wanted to take the naloxone training (i.e. required by employer, would like to know how to save a life, and other).

### Pre-and post-test assessments

The pre-and post-test included 29 questions assessing participants’ knowledge, confidence, and attitudes (See Appendix A). The 20 knowledge questions were modified using questions from the previously validated Opioid Overdose Knowledge Scale and were categorized under the four subcomponent composite scores of naloxone administration (n = 14), risk factors (n = 9), overdose signs (n = 12), and overdose response scenarios (n = 8). A score of one was given for each correct response and zero if the response was incorrect; therefore, the possible total knowledge composite score ranged between 0 and 43.

The five confidence questions were based on a 5-point Likert scale (1 = strongly disagree to 5 = strongly agree) that assessed LEO’s confidence in recognizing signs and symptoms of an overdose, responding to an overdose, administering naloxone, training others how to use naloxone, and knowing what to do after giving naloxone (Dahlem et al., 2020). The confidence scores ranged from 5 to 25.

Lastly, LEOs’ attitudes and perceptions towards naloxone and PWUD were assessed for if the participant believed administering naloxone enabled the person to continue to use more drugs, if using naloxone is a waste of resources because the person might continue to use drugs, if they feared harming someone when administering naloxone, and if they believed using naloxone will delay entry into drug treatment. The four attitude questions were based on a 5-point Likert scale (1 = strongly disagree to 5 = strongly agree) with a possible total composite score that ranged from 4 to 20. After the LEOs completed the training, they took a post-test comprised of the same items and scale scores.

### Data analysis

All data management was carried out using the software SAS 9.4; Statistical analyses were conducted in Stata 17. Seven separate models were constructed for each sub-scale embedded in the pre/post questionnaire: total knowledge, confidence, and attitude composite score and a model for each of the major subcomponents of the total knowledge score including naloxone administration, risk factors, overdose signs, and scenario response. Given our evaluation of the distributional properties of each outcome, we evaluated the effects of the training on total knowledge and its sub-component scores using a mixed-effects Poisson statistical model with a fixed effect term comparing pre vs. post and a random Y-intercept to accommodate the repeated measures nested within LEO officer. The variance for the total attitude composite score was more appropriately analyzed using a similarly designed mixed-effects negative binomial model.

## Results

### Demographic characteristics

From May 2019 to September 2020, 198 completed the pre-test survey and 186 completed the post-test survey. A total of 182 LEOs from the five-law enforcement agencies were included in the analysis based on completeness of both the pre-test survey and post-test survey, representing 92% of the LEO cohort. Table 1 depicts the demographic characteristics collected during the study. We utilized Somer’s D to evaluate the associations between gender and race and the outcomes of knowledge, confidence, attitude, and knowledge subcomponents of naloxone administration, risk factors, overdose signs, and scenario response composite scores. The Somer’s D did not reveal significant associations between any of the outcomes and either gender or race.

**Table 1:** Demographic characteristics

Gender	*N*	*%*
Male	140	76.92
Female	37	20.33
Transgender	1	0.55
I prefer not to say	4	2.20
**Race/Ethnicity**	*N*	*%*
White	156	85.71
African American	11	6.04
Asian	2	1.1
Hispanic/Latino	4	2.2
Native American	1	0.55
Other	8	4.40
**Ethnicity**	*N*	*%*
Non-Hispanic	178	97.8
Hispanic	4	2.2
**Law Enforcement Agency**	*N*	*%*
A	12	6.63
B	76	41.99
C	62	34.25
D	23	12.71
E	8	4.42
**Have Used Naloxone Previously**	*N*	*%*
Yes	12	6.6
No	133	73.08
Not Reported	37	20.32
**Number of Times Naloxone Used in the Field**	*N*	*%*
0	133	73.08
1–9	11	6.05
10–100	1	0.55
Not Reported	37	20.32
**Age**	*Mean (SD)*	*Range*
	36 (10.6)	18–62

### Composite scores pre- versus post-training

For the total knowledge composite score including its subcomponents and the confidence composite scores, higher scores indicated increased knowledge and confidence. Conversely, the attitude questions were negatively worded, so a higher score indicated more negative attitudes towards naloxone and addiction. There was a significant increase in total knowledge composite score after receiving the web-based naloxone training (*p < 0.01*, Cohen’s D = 0.56), with the median (IQR) pre-training score of 35 (32, 37) increasing to 40 (39, 42) (Table 2). There was also a significant increase after training in the total confidence composite score (*p < 0.01*,Cohen’s D = 0.47), with the pre-training median (IQR) of 18.5 (15, 20) and post-training median of 20 (20, 25). However, there was no significant change in total attitude composite score after receiving training (*p = 0.80*, Cohen’s D = 0.02). The median (IQR) attitude score pre-training was 8 (6, 10) and post-training remained 8 (4, 10).

**Table 2:** Poisson and negative binomial regression results of web-based naloxone training for law enforcement officers

	Knowledge Composite Score	Confidence Composite Score	Attitude Composite Score
	N = 309^a^	N = 354^a^	N = 354^a^
	IRR (95% CI)	IRR (95% CI)	IRR (95% CI)
Time			
Before Training	Ref	Ref	Ref
After Training	1.16 (1.12, 1.21)	1.16 (1.11, 1.22)	0.99 (0.92, 1.07)
Cohen’s D	0.56	0.47	0.02

**Table 2.** Reference group: Time = Before Training

a. An observation with any missing variables was dropped from the model.

Three out of four subcomponents of the total knowledge composite score, naloxone administration, risk factors, and overdose signs, also saw a significant increase after training (Table 3). The median naloxone administration composite score increased from 12 (10, 13) to 13 (13, 14) (*p < 0.01*, Cohen’s D = 0.31). The median risk factors composite score increased from 7 (7, 8) to 8 (8, 9) *(p = 0.01*, Cohen’s D = 0.25). The median for the overdose signs composite score increased from 8 (6, 9) to 11 (10, 12) after training (*p < 0.01*, Cohen’s D = 0.62). The was no significant change in the scenario response composite score. The possible range for that score was 0 to 8 and even before training the median (IQR) score amongst LEOs was high, going from 7 (7, 8) to 8 (7, 8) after training (*p = 0.08*, Cohen’s D = 0.13).

**Table 3:** Poisson regression results of web-based naloxone training for law enforcement officers-subcomponents of knowledge composite score

	Naloxone Administration Composite Score	Risk Factors Composite Score	Overdose Signs Composite Score	Scenario Response Composite Score
	N = 315^a^	N = 362^a^	N = 362^a^	N = 350^a^
	IRR (95% CI)	IRR (95% CI)	IRR (95% CI)	IRR (95% CI)
Time				
Before Training	Ref	Ref	Ref	Ref
After Training	1.15 (1.07, 1.23)	1.13 (1.05, 1.22)	1.33 (1.24, 1.42)	1.07 (0.99, 1.16)
Cohen’s D	0.31	0.25	0.62	0.13

**Table 3.** Reference group: Time = Before Training

a. An observation with any missing variables was dropped from the model.

## Discussion

To our knowledge, this is the first study to show the direct effects of web-based naloxone training for LEOs on knowledge, confidence, and attitudes. We found that our web-based naloxone training of LEOs had a significant effect on increasing LEOs overall overdose knowledge and confidence in training others, in recognizing the signs of overdose and responding to overdoses. However, the web-based naloxone training did not impact LEO’s attitudes and perceptions towards naloxone and PWUD.

Our positive improvements in knowledge and confidence are consistent with other studies that looked at in-person first responder naloxone training (Crocker et al., 2019; Saucier et al., 2016; Wagner et al., 2016) and online training for first responders (Simmons et al., 2016) and first-year medical students (Berland et al., 2019). In particular, Berland et al. (2019) found that the educational outcomes for first year-medical students were not statistically different for the knowledge, preparedness, and attitudes factors when comparing online and in-person opioid overdose prevention training (Berland et al., 2019). Overall, we can see that knowledge and confidence increase in both in-person and online training settings.

Although our web-based training included additional topics that addressed misconceptions about naloxone and PWUD through LEO and people in long-term recovery testimonials (Dahlem et al., 2022), these videos were not sufficient to produce a change in attitudes towards naloxone and PWUD. This is consistent with Winograd et al. study (2020a) where 31% of the LEOs held more negative attitudes and 13.7% had no attitude changes toward naloxone and overdose survivors following a comprehensive overdose education and naloxone distribution training (Winograd et al., 2020a). The worsening of attitudes was particularly true for younger LEOs (Winograd et al., 2020a).

We were unable to assess the reasons behind the lack of change in attitudes of LEOs after our web-based training. Studies have shown that LEOs generally hold a negative view of PWUD(Ezell et al., 2021; Reichert et al., 2023) and have misconceptions about fentanyl (Attaway et al., 2021). Negative attitudes toward naloxone were also found among a sample of first responders, particularly for those who did not view addiction as a chronic disease, lived in rural areas, had previously administered naloxone, and had opioid use disorder stigma (Kruis et al., 2022). However, in Wagner’s et al. study (2016), LEOs attitudes toward overdose survivors did not change based on naloxone training but the LEOs expressed positive experiences and changes in attitudes towards naloxone after using naloxone in the field. These feelings of goodwill were also expressed in interviews with LEOs who had used naloxone in the field and experienced a successful resuscitation (Dahlem et al., 2022). Future research could consider using a larger sample with a validated tool to examine LEOs attitudes before and after overdose reversal.

Irrespective of whether LEO attitudes changed or not due to the naloxone training, studies show the importance of naloxone training for LEOs to be equipped with naloxone, and LEOs save lives. LEOs are often first to respond to overdoses in advance of emergency medical personnel (White et al., 2022) particularly in rural settings (Wood et al., 2021) and they fulfill their duties (Dahlem et al., 2022). LEOs who are naloxone trained are more likely to respond to at least one overdose in the past six months than LEOs who are not trained (Carroll et al., 2020). Moreover, other studies have shown that trained LEOs effectively recognize and respond to overdose events after naloxone training (Dahlem et al., 2017; Fisher et al., 2016; Pourtaher et al., 2022; Wagner et al., 2016; White et al., 2022). Being first to arrive at the scene of overdose is important as to reduce complications associated with a hypoxic brain. These studies demonstrate that LEOs administer naloxone regardless of their attitudes about naloxone and PWUD, and do play an important role in reducing opioid overdose deaths.

However, the role of police involvement in overdose response has been highly debated among harm reductionists and advocates for PWUD (del Pozo, 2022; Doe-Simkins et al., 2022). These unintended consequences of involving the police have been related to limitations in Good Samaritan Laws that lead to arrests of overdose survivors and those who call for emergency services (Carroll et al., 2020; Koester et al., 2017; White et al., 2022), drug induced homicide charges (Carroll et al., 2021), history of negative interactions with the police and distrust related to perceptions of police conduct during overdoses (Latimore & Bergstein, 2017; van der Meulen & Chu, 2022; van der Meulen et al., 2021). Therefore, it is important that when LEOs respond to an overdose, that the scene is considered a medical emergency, not a criminal investigation, and relational efforts made to restore the negative interactions with the police and the community.

Training law enforcement to respond to overdoses is one method to increase naloxone access. However, PWUD, their social networks, and other community laypeople are the official first responders as they are the ones to initiate the 911 response. In addition, modeling studies have shown that naloxone distribution to community laypeople are associated with maximum health gains and to be most cost effective (Townsend et al., 2020; Coffin & Sullivan, 2013). Despite continued efforts to increase public access to naloxone, a recent modeling study found that all states except for Arizona had underdeveloped naloxone distribution, and few were able to avert 80% of witnessed overdose deaths with naloxone (Irvine et al., 2022). Therefore, policies and resources should be prioritized to expand community naloxone distribution particularly for PWUD and those who are more likely to be present at an overdose (del Pozo, 2022; Doe-Simkins et al., 2022). Notwithstanding, other community interventions such as non-police overdose response teams for mental health crisis are growing in popularity and could be an alternative overdose response model to mitigate the fears associated with arrests at an overdose, limitations to the Good Samaritan Laws, and mistrust of police (Enos, 2020; Marcus & Stergiopoulos, 2022).

This study further supports a need for comprehensive addiction curriculum for LEOs that specifically addresses stigma, harm reduction services, and disease of addiction. Promising curriculum such as the Safety and Health Integration in the Enforcement of Laws on Drugs (SHIELD) program may be an avenue to facilitate greater addiction and harm reduction knowledge for LEOs (Beletsky et al., 2021). The SHIELD training addresses one notable misconception that is commonly found in the news media with LEOs falsely believing they can experience a fentanyl overdose simply through incidental contact (del Pozo et al., 2021). Our web-based naloxone training addressed misconceptions about fentanyl overdoses through incidental contact that has been heightened by social media (Beletsky et al., 2020) and provides guidance on steps to take when exposed to unknown substances (i.e. wear gloves, do not smell or touch unknown substances, wash hands with soap and water). In addition, we include position statements from American College of Medical Toxicology (Moss et al., 2017) indicating incidental contact is unlikely to cause opioid toxicity. However, we did not specifically measure knowledge effects regarding misconceptions about fentanyl overdoses in LEOs when responding to overdose. Future research can include direct measurements about fentanyl misconceptions that are addressed in naloxone trainings. Future trainings should specifically add another misconception that “naloxone is for the police due to incidental contact of fentanyl exposure when responding to overdoses.” Police are equipped with naloxone because they are often the first responder to arrive at the scene of an overdose and there should not be any hesitation in responding to an overdose due to fears of fentanyl exposure. This misconception of fears of fentanyl exposure when responding should be emphasized in all overdose training curriculum.

Another stigma intervention, ArtSpective, uses art and museum pedagogy to reduce stigma towards perinatal opioid use among nursing students and midwives which can be translated to LEOs (Shuman CJ et al., in press). A recent study showed their Opioid Overdose Awareness and Reversal Training (OOART) curriculum was effective in reducing stigmatizing attitudes toward people with opioid use disorder across a diverse population, but was predominantly young students in health-related fields (Bascou et al., 2022).

Stigma reduction education should include providing information on disease of addiction, exposing LEOs to people who are in recovery or stories of recovery, and correcting misinformation on social media about fentanyl (Kruis et al., 2020, 2022). In a Midwest sample of LEOs, Reichert et al. (2023) found that majority of LEOs held stigmatizing views of distrust, blame, shame, and fear toward people with opioid use disorders, and various LEO characteristics such as gender, race/ethnicity, education, and work setting were associated with higher stigmatizing attitudes. Though validated surveys that directly measure police stigma are still needed, further training and educating LEOs on addiction, treatment, and hearing personal recovery stories may reduce stigma associated with PWUD (Reichert et al., 2023). Therefore, future research should investigate the underlying mechanisms of these negative attitudes and utilize multi-prong educational approaches to reduce stigma in LEOs about PWUD and naloxone.

### Limitations

Although the web-based naloxone training had positive effects on knowledge and confidence, some study limitations exist. Our study included only five law enforcement agencies with participating LEO’s varying from n = 8 to n = 76. All law enforcement offices were located in a predominantly urban Midwestern region of the United States which limits the generalizability of our results, and our ability to address possible office-level effects that may have impacted the training effectiveness. Moreover, we did not use a validated attitude scale to measure LEOs’ attitudes toward naloxone and PWUD. We did not collect overdose response outcomes for LEOs who were trained through our web-based training. Future research can examine the effects of overdose response for LEOs who were trained in person vs. those who were trained online. Future research should use a validated attitude measure that directly assesses stigma and relationships among knowledge, attitudes, and behaviors. Another limitation is the response bias that may have occurred while using a self-report survey. All LEOs had the opportunity to enroll into the naloxone training anytime throughout their working shift. We did not collect the information on when each participant accessed the site and the length of time it took them to complete the training. However, our prior feasibility study indicates a median completion time of 45 min (range 37–80 min). It is possible that the LEOs may have experienced respondent fatigue after going through other trainings or working a third shift.

## Conclusion

Our web-based naloxone training had a significant impact on LEO’s overdose knowledge and confidence in responding to overdoses and administering naloxone. Using a web-based format has the potential to reach a wider audience and advances dissemination of this life-saving intervention to assist LEOs to respond to overdoses. Additional curriculum expansion is needed to specifically address the attitudes towards naloxone and PWUD.

### Electronic supplementary material

Below is the link to the electronic supplementary material.

Supplementary Material 1

Supplementary Material 2

## Data Availability

The datasets generated and analyzed are not publicly available due to ongoing analyses by the authors. However, de-identified data supporting the findings of this study are available from the corresponding author upon reasonable request.

## Abbreviations

LEO: Law Enforcement Officers
IQR: Interquartile Range
SHIELD: Safety and Health Integration in the Enforcement of Laws on Drugs
OOART: Opioid Overdose Awareness and Reversal Training
