# Supplementary material for: Effectiveness of take ACTION online naloxone training for law enforcement officers
Source: Health Justice. 2023 Nov 18;11:47. doi: 10.1186/s40352-023-00250-9 (PMC10656891; doi:10.1186/s40352-023-00250-9)
Supplement: Supplementary file 1 — Supplementary Material 1 [file 40352_2023_250_MOESM1_ESM.docx]

**Appendix**

**Knowledge Measures***

**1. When using intranasal naloxone (Narcan), you should do a test spray. (True or False)**

1) True
2) False
3) Don't know

**2. If the first dose of naloxone has no observable effect after 2-3 minutes, a second dose can be given.  (True or False)**

1) True
2) False
3) Don't know

**3. Naloxone may cause withdrawal symptoms. (True or False)**

1) True
2) False
3) Don't know

**4. Although the risk is low, a person can overdose again after having received naloxone.  (True or False)**

1) True
2) False
3) Don't know

**5. Naloxone's duration (how long it lasts in the body) is shorter than the duration of heroin. (True or False)**

1) True
2) False
3) Don't know

**6. Identify which of the following is NOT an opioid**

1) Heroin

2) Fentanyl

3) Methadone

4) Alprazolam

**7. Incidental skin contact with fentanyl occur during daily activities. Harmful effects are not expected if the contaminated skin is promptly washed off with soap and water**

1) True
2) False
3) Don't know

**8. After administering naloxone, the officer should:**

1) Follow the referral process to treatment and further medical care if established within your county
2) Complete the appropriate police documentation
3) All of the above

**9. In an opioid overdose, the most dangerous side effect of taking too many opioids is…**

1) Respiratory Depression
2) Headache
3) Nausea and Vomiting
4) Tremors

**10.What is naloxone used for?**

1) To reverse the effects of an opioid overdose (heroin, fentanyl, methadone, etc.)
2) To reverse the effects of a methamphetamine overdose
3) To reverse the effects of a cocaine overdose
4) To reverse the effects of any overdose
5) Don’t know/not sure

**11. How long does naloxone take to start having an effect?**

1) 2-5 minutes
2) 6-10 minutes
3) 11-20 minutes
4) 21-40 minutes
5) Don’t know/not sure

**12. How long do the effects of naloxone last for?**

1) Less than 20 minutes
2) 30-120 minutes
3) 1 to 6 hours
4) 6 to 12 hours
5) Don’t know/not sure

**13. Michigan legislation protects you from civil and criminal liability in the event you administer naloxone in good faith to someone you suspect is experiencing an opioid overdose.**

1) True
2) False
3) Don't know

**14. The Good Samaritan Law protects the overdosing drug user from possession charges and illicit use of controlled substances when seeking help for him/herself or calling for medical assistance for someone else.**

1) True
2) False
3) Don't know

**15. Which of the following factors increase the risk of a heroin (opioid) overdose? (select all that apply)**

1) Prior history of an overdose

2) Using drugs with friends

3) Transition from oral use to injection use

4) Higher purity of the drug

5) Age greater than 40 years

6) Mixing drugs such as alcohol and anti-anxiety medications with opioids

7) Prior criminal history

8) Changes in tolerance (such as recently discharged from jail or detox facility)

9) Education less than high school

**16. Which of the following are signs and symptoms of an opioid overdose? (Select all that apply)**

1) Slow, erratic, absent breathing

2) Bluish purple lips and fingernails; ashen grey if darker skinned

3) Unresponsive to pain stimulus

4) Seizures

5) Pupil dilation

6) Choking sounds, or snore-like gurgling noises

7) Unconscious

8) Agitated behavior

9) Bloodshot eyes

10) Pinpoint pupils

11) Rapid heartbeat

12) Profuse sweating

**17. You come upon a person who is stumbling while he walks and is obviously high. You should administer naloxone.**

1) Correct

2) Incorrect

3) Don’t know

**18. You come upon a person turning blue on a park bench with very slow breathing. Should you administer naloxone?**

1) Correct

2) Incorrect

3) Don’t know

**19. You are called to a domestic dispute and the wife says, “He did some drugs and now he is aggressive and going crazy.”  After he is secured, should you give him naloxone?**

1) Correct

2) Incorrect

3) Don’t know

**20. When encountering an unknown substance (white powder, pills, capsules, blotted paper) at the scene, you should (check all that apply)**

1) Touch the unknown substance with bare hands

2) Sniff the unknown substance to check out the smell

3) Taste the unknown substance to see if it's powdered sugar

4) Be aware of the environment

5) Always wear gloves, minimize skin contact, and avoid actions that may cause powder to become

airborne. If needed, wear other personal protective equipment, such as eye protection and approved

N95 or P100 respirator mask.

**Confidence Measures**

**1. I am confident that I can recognize signs and symptoms of an opioid overdose (Note: there is no "wrong" answer).**

1) Strongly Disagree
2) Disagree
3) Neutral
4) Agree
5) Strongly Agree

**2. I am confident that I know how to respond to an opioid overdose (Note: there is no "wrong" answer).**

1) Strongly Disagree
2) Disagree
3) Neutral
4) Agree
5) Strongly Agree

**3. I am confident that I know how to give naloxone (Note: there is no "wrong" answer).**

1) Strongly Disagree
2) Disagree
3) Neutral
4) Agree
5) Strongly Agree

**4. I am confident that I can train others in how to use naloxone (Note: there is no "wrong" answer).**

1) Strongly Disagree
2) Disagree
3) Neutral
4) Agree
5) Strongly Agree

**5. I am confident that I know what to do after giving naloxone (Note: there is no "wrong" answer).**

1) Strongly Disagree
2) Disagree
3) Neutral
4) Agree
5) Strongly Agree

**Attitudes Measures**

**1. If I administer naloxone, I am enabling the person to continue to use more drugs (Note: there is no "wrong" answer).**

1) Strongly Disagree
2) Disagree
3) Neutral
4) Agree
5) Strongly Agree

**2. It is a waste of resources to save a person with naloxone since the person will overdose again and again. (Note: there is no "wrong" answer).**

1) Strongly Disagree
2) Disagree
3) Neutral
4) Agree
5) Strongly Agree

**3. I am afraid that I may cause harm if I use naloxone on someone (Note: there is no "wrong" answer).**

1) Strongly Disagree
2) Disagree
3) Neutral
4) Agree
5) Strongly Agree

**4. I believe using naloxone will delay entry into drug treatment (Note: there is no "wrong" answer).**

1) Strongly Disagree
2) Disagree
3) Neutral
4) Agree
5) Strongly Agree

*20 questions of **KNOWLEDGE** based on questions 1 to 20**.**

- Questions 1-14 pertain to **NALOXONE ADMINISTRATION (score out of 14)**
- Question 15 pertain **RISK FACTORS (score out of 9) – for each of the 9 options that the law enforcement officers correctly selected or correctly did not select, they gained one point, thus a final score out of 9**
- Question 16 pertain to overdose **SIGNS/SYMPTOMS (score out of 12) – for each of the 12 options that the law enforcement officers correctly selected or did not select, they gained one point, thus a final score out of 12**
- Questions 17-20 pertain to **SCENARIO RESPONSE (score out of 8) – for each of the 4 options that the law enforcement officers correctly selected or correctly did not select, they gained one point. This coupled with questions 17-19 results in a final score out of 8**

5 questions of **CONFIDENCE**

4 questions of **ATTITUDES**
