# Supplementary material for: Effectiveness of take ACTION online naloxone training for law enforcement officers
Source: Health Justice. 2023 Nov 18;11:47. doi: 10.1186/s40352-023-00250-9 (PMC10656891; doi:10.1186/s40352-023-00250-9)
Supplement: Supplementary file 2 — Supplementary Material 2 [file 40352_2023_250_MOESM2_ESM.docx]

| **Reviewers’ Comments** | **Author’s Response** |
| --- | --- |
| Reviewer #1: Important study. Overdoes are climbing in US. LEOs are the best frontline administrators of the antidote. | Thank you for taking the time to review the paper and for supporting our work. |
|  |  |
| Reviewer #2: This revision did a very good job of answering my original observations. My remaining points are therefore minor: | Thank you for providing another thorough review of the paper. |
| 1) Please explicitly tie the discussion about how highly contested police overdose response is with this presumption in your training curriculum: "4) Since an overdose is a medical issue, EMS should respond rather than law enforcement." Many would say this is highly debatable, given concerns about arrests, etc. at the scenes of an overdose. On the other hand, police get there first in many cases, as you now note. A few sentences that tie this together in the discussion and bring it explicitly back to this aspect of the training would solidify the paper, considering this aspect of the training will be contested by many. Minor revisions and tying what you have already written together should do. | Thank you for this comment. I have highlighted in bold the additions made to tie these thoughts together.  **Being first to arrive at the scene of overdose is important as to reduce complications associated with a hypoxic brain. T**hese studies demonstrate that LEOs administer naloxone regardless of their attitudes about naloxone and people who use drugs, and do play an important role in reducing opioid overdose deaths.  However, the role of police involvement in overdose response has been highly debated among harm reductionists and advocates for people who use drugs (del Pozo, 2022; Doe-Simkins et al., 2022). These unintended consequences of involving the police have been related to limitations in Good Samaritan Laws that lead to arrests of overdose survivors and those who call for emergency services (Carroll, Mital, Wolff et al., 2020; Koester, Mueller et al., 2017; White et al., 2022), drug induced homicide charges (Carroll, Ostrach et al., 2021), history of negative interactions with the police and distrust related to perceptions of police conduct during overdoses (Latimore & Bergstein, 2017; van der Meulen & Ka Hon Chu, 2022; van der Meulen, Ka Hon Chu, Butler-McPhee, 2021). **Therefore, it is important that when LEOs respond to an overdose, that the scene is considered a medical emergency, not a criminal investigation, and relational efforts made to restore the negative interactions with the police and the community.** |
| 2) Please tie debunking the fentanyl exposure myth more discretely to the purpose of training police on naloxone administration; it seems like a necessary component of a holistic approach. Many police say they carry naloxone first and foremost to reverse overdoses they and other police may experience after exposure to fentanyl, and there is the worry that this fear will chill overdose response and naloxone administration. Effective police training needs to overcome the belief naloxone is carried by police for police, and there is reason to hesitate at the scene of an OD for fear of exposure. If that wasn't covered in the training, consider emphasizing the need for it in future or other training modules. That was the purpose of requesting mention in the prior review. | We have added to the text on pg 14-15.  **“Future trainings should specifically add another misconception that “naloxone is for the police due to incidental contact of fentanyl exposure when responding to overdoses.” Police are equipped with naloxone because they are often the first responder to arrive at the scene of an overdose and there should not be any hesitation in responding to an overdose due to fears of fentanyl exposure. This misconception of fears of fentanyl exposure when responding should be emphasized in all overdose training curriculum.** |
| 3) it does not appear that you have updated your reference list. | Sincere apologies for this error. The references have been updated. |
| 4) Consider using PWUD after the first mention of people who use drugs. | PWUD has been added following the initial mention of people who use drugs. |
| Thank you again for the opportunity to review this paper. | Thank you kindly for another thorough review of the paper. |
